# Relationship between Mental Health, the *CLOCK* Gene, and Sleep Quality in Surgical Nurses: A Cross-Sectional Study

**DOI:** 10.1155/2020/4795763

**Published:** 2020-08-29

**Authors:** Lingyun Shi, Yuanyuan Liu, Ting Jiang, Ping Yan, Fan Cao, Ying Chen, Huanhuan Wei, Jiwen Liu

**Affiliations:** ^1^Joint Surgery Department, The First Affiliated Hospital of Xinjiang Medical University, Urumqi 830054, China; ^2^Disinfection and Distribution Center of the First Affiliated Hospital of Xinjiang Medical University, Urumqi 830054, China; ^3^Department of Public Health, Xinjiang Medical University, Urumqi 830011, China; ^4^Department of Nursing, Xinjiang Medical University, Urumqi 830011, China

## Abstract

Nursing is a high-risk occupation with high exposure to stress. The physical and mental health of nurses is directly related to the quality of medical services. Therefore, the sleep quality of nurses should not be ignored. In this study, the method of cluster random sampling was adopted from May to September 2019, and a questionnaire survey was conducted among 521 surgical nurses from five affiliated hospitals of Xinjiang Medical University. The relationship between mental health and sleep quality was analyzed, and 20% of the participants with sleep disorders were randomly selected. The sleep disorders used 1 : 1 matching, finally providing a sample with 60 cases and 60 controls for measurement of the *CLOCK* gene (rs1801260, rs6850524), to analyze the effect of the interaction between mental health and the *CLOCK* gene on sleep. The mental health and sleep quality of the surgical nurses were evaluated using the Symptom Checklist 90 (SCL-90) and Pittsburgh Sleep Quality Index (PSQI). The study found that surgical nurses had poor sleep, and there were differences associated with age, years working, frequency of night shifts, and incidence of sleep disorders under marital status (*p* < 0.05). The PSQI scores of the positive psychological symptoms were higher than those of the negative psychological symptoms. The rank sum test was used to compare the sleep quality scores of different genotypes in *CLOCK* rs1801260 and rs6850524; the results indicated that the PSQI scores were different among different genotypes at the rs1801260 and rs6850524 loci. The logistic regression results suggested that *CLOCK* gene rs1801260 (TC) and positive psychological symptoms were influential factors for sleep disorders, and the interaction of positive psychological symptoms∗rs1801260 (TT) was a risk factor for sleep disorders (OR = 10.833, 95% CI: 2.987–39.288). The sleep quality of nurses is not only affected by demographic characteristics but also affected by mental health status and the *CLOCK* gene.

## 1. Introduction

The incidence of sleep problems is increasing year by year, and sleep disorders have become a common clinical disease [1]. Sleep problems are a risk factor for a variety of mental health and chronic diseases [2]. The occurrence of sleep problems reduces the quality of life and working ability of professional people and greatly increases the risk of accidents at work. At the same time, the economic loss caused by sleep problems is as high as US $100 billion every year [3]. Lack of sleep or poor quality sleep can lead to abnormal self-regulatory functions of the body and disorder of the circadian rhythm of the biological clock. Negative emotions such as anxiety and depression can be generated during normal life, and fatigue, burnout, and slowdown can occur in the working life [4–6].

The particularity of the medical service industry leads to an increased risk of sleep disorders among medical workers [7]. The sleep condition of nursing staff is not favorable, and the detection rate of sleep problems is much higher than that in the general population, mainly manifested as insomnia, less effective sleep time, and low sleep quality [8, 9]. Many studies on sleep quality have shown that there is a very close relationship between population characteristics, working conditions, and living habits [10, 11]. The working environment, work content, and workload of different departments also have a significant impact on the sleep quality of medical staff [12]. Also, the proportion of mental abnormalities and psychological problems associated with sleep disorders is quite high, usually manifested as depression, anxiety, and irritability [13–15].

Mental disorder refers to the mental illness caused by negative emotions or behavioral deviations following various adverse stimuli [16]. When people are under psychological stress, they will not only react in an emotional way but also reflect it through the physical body [17]. That is to say, when people face pressure for a long time, the body will produce a variety of uncomfortable symptoms, such as dizziness and poor sleep. A number of studies have shown that sleep is easily affected by mood and that positive psychological symptoms can reduce sleep quality [18–21]. Duncan et al. and Pilcher et al. [22, 23] believe that mental health is related to regular sleep, while emotional stress, depression, anger, and confusion are more related to the quality of sleep.

Studies have shown that sleep disorders may involve genetic factors, including *CLOCK* genes [24]. The *CLOCK* gene is the first biological gene to be discovered that maintains the circadian rhythms of physiology and behavior, and it is an important component of the positive feedback regulation of biological *CLOCK* rhythm [25]. It has been found that hypomethylation of the *CLOCK* gene promoter and high expression of the *CLOCK* gene occur in the peripheral blood of long-term night shift workers, which has further demonstrated the relationship between *CLOCK* gene regulation and circadian rhythm and has also provided evidence for the epigenetic regulation of biological rhythms [26–29]. Kripke et al. [30] found that polymorphisms of the *CLOCK* gene, rs3805148 and rs12504300, were associated with the incidence of bipolar depression disorder. Meanwhile, polymorphisms rs11932595 and rs3805148 of the *CLOCK* gene were also found to be associated with sleep type and sleep disorder in patients with single or bipolar depression [31–33]. However, there are also different conclusions, for example, that the *CLOCK* gene can affect sleep but is not associated with depression [34]. Current studies suggest that mental health and *CLOCK* genes are all factors affecting sleep, but the effect of the interaction between mental health and the *CLOCK* gene on sleep is inconsistent.

Surgical nursing requires high professional technical requirements, complex work content, and high work intensity. Surgical nurses are more likely to have mental health problems and sleep problems due to increased stress in their daily work. It was found that the distribution of *CLOCK* gene rs1801260 and rs6850524 was different in the positive psychological symptom group and the negative psychological symptom group [35]. Therefore, surgical nurses were selected as research subjects to investigate their mental health status, and the rs1801260 and rs6850524 sites of the *CLOCK* gene were measured in this study, to analyze the influence of mental disorder and the *CLOCK* gene on sleep and to explore the influence of the interaction between mental disorder and the *CLOCK* gene on sleep.

## 2. Materials and Methods

### 2.1. Participants

This study was conducted in five affiliated hospitals of Xinjiang Medical University, China. Consent was obtained after communication with each hospital prior to the investigation. The survey was carried out from May to September 2019. Surgical nurses from the five affiliated hospitals were investigated in this study. The surgery mainly included cardiothoracic surgery, neurosurgery, hepatobiliary surgery, urology surgery, anorectal surgery, burn surgery, breast surgery, pediatric surgery, plastic surgery, orthopedics, and hand surgery, which amounted to a total of 11 departments, and each department employed an average of 10–15 nurses. Using the cluster random sampling method, ten nurses were randomly selected from each department for investigation. A list of personnel from each department was obtained from the chief nurse of each department and, according to the list, the research participants were randomly selected. The inclusion criteria were the following: (1) age: 18–60 years, (2) continuous working time ≥ 0.5 years, and (3) registered nurse of clinical practice. A total of 550 questionnaires were distributed, and 541 questionnaires were retrieved. Thereafter, 521 valid questionnaires were finally retrieved, with an effective recovery rate of 94.73%. The research design was approved by the ethics committee of Xinjiang Medical University.

In this study, a total of 298 people with positive sleep disorders were screened. The general indicators were measured with 10%–20% of the randomly selected questionnaire responders as experimental research participants. Therefore, research on sleep disorders involved 20% positive participants randomly chosen for the experimental research; the sleep disorder study used 1 : 1 matching, and participants negative for sleep disorders who were of the same ethnicity and age ± 1 years were selected as controls for the case–control study, finally providing a sample with 60 cases and 60 controls.

### 2.2. Measures

#### 2.2.1. Mental Health Status

Symptom Checklist 90 (SCL-90), compiled by Derogatis in 1975 [36], was used; the scale was translated and introduced into China in 1986, and it was proven to have good reliability and validity [37]. The scale includes 9 factors and a total of 90 evaluation items. The 1–5 scoring method was adopted, and the total score and factor score were used as the indicators to evaluate the mental health status (i.e., if the total score ≥ 160 points, the score of any factor is >2, or the number of positive items is >43, this indicates that there may be a mild or greater level of psychological symptoms; that is, there may be mental health problems) [38].

#### 2.2.2. Sleep Quality

The Pittsburgh Sleep Quality Index (PSQI) compiled by Dr. Buysse et al. in 1989 was used [39]. The Chinese version of the questionnaire (CPSQI) has good reliability and validity after testing [40]. The scale consists of 7 subitems, each of which is graded from 0 to 3. The sum of the 7 items is the final score of the PSQI (0 to 21 points). According to the scoring criteria, a total score on the PSQI > 7 is considered to represent sleep disorder [41].

### 2.3. Genotyping

Venous blood samples were collected in EDTA-containing tubes from all participants following a 12 h fast. Genomic DNA was purified from the samples using a whole blood genome extraction kit (Tiangen Biotech, Beijing, China) and cryopreserved at −20°C until use. The SNPs rs1801260 and rs6850524 were genotyped with the SNaPshot SNP assay using the primers listed in Table 1. Data were analyzed using GeneMapper 4.1 (Applied Biosystems, Foster City, CA, USA). For quality control, 10% of randomly selected samples were genotyped a second time by different researchers, yielding 100% reproducibility.

### 2.4. Quality Control

#### 2.4.1. Quality Control of Field Questionnaire Survey

The investigators were trained to ensure the quality and progress of the investigation. Interviewers in the field survey were required to elaborate on the content of the questionnaire and the purpose of the research to the respondents before issuing the questionnaire during the survey, and the respondents were required to volunteer to send the questionnaire to them. They assisted the respondents to fill in the questionnaire accurately and completely.

#### 2.4.2. Laboratory Quality Control

Blood samples were collected by professional medical personnel. The experimental process strictly followed the quality control standards of the laboratory. DNA extraction and SNP detection were carried out in strict accordance with the kit instructions. A blind method was adopted for genetic testing, and samples were randomly selected at 10% to check the consistency of the results. When the results of the retest were inconsistent with the initial test, the experiment was repeated to check again.

### 2.5. Statistical Analysis

SPSS for Windows v.22.0 software (SPSS Inc., Chicago, IL) was used for data processing and statistical analysis. The measurement data were statistically described using $\overset{¯}{\chi }\pm S$, the mean values of the two groups were compared using the *t*-test for two independent samples, and the mean values of multiple groups were compared using one-way analysis of variance. As the PSQI index value does not follow a normal distribution, a nonparametric test is used. If there were differences in the whole population, the *SNK-q* and *LSD* tests were used for pairwise comparison, and the rates were compared using the *chi-squared* test. The significance level was *α* = 0.05.

## 3. Results

### 3.1. Sleep Disorders in Nurses with Different Demographic Characteristics

The chi-squared test was used to compare the incidence of sleep disorders under different demographic characteristics. The results indicated that the incidence of sleep disorders under different demographic characteristics could be considered as different with age (*p* = 0.031), working age (*p* = 0.038), night shift frequency (*p* < 0.001), and marital status (*p* = 0.027). Among different ages, the linear trend test showed that the detection rate increased with the increase of age, but this trend was mainly reflected in the group under 25 years old and the group between 26 and 35 years old, with little change after 35 years. Among the different age groups, the incidence of sleep disorders in nurses with working age less than 10 years was lower (53.3%), and the incidence of sleep disorders in nurses with working age more than 10 years was higher (62.7%). The incidence of sleep disorders in nurses with shift frequency ≤ 3 times/month was lower (29.8%), while the incidence of sleep disorders in the group with shift frequency > 3 times/month was higher (66.4%). The incidence of sleep disorders was higher in the married group (60.1%) than in the single group (49.3%) (Table 2).

### 3.2. Relationship between Mental Health and Sleep Quality

The rank sum test was used for analysis, and the results showed differences for the PSQI score (*p* < 0.001), subjective sleep quality (*p* < 0.001), time of falling asleep (*p* < 0.001), sleeping time (*p* = 0.002), sleep efficiency (*p* < 0.001), sleep disorders (*p* < 0.001), hypnotic drugs (*p* = 0.047), and daytime dysfunction (*p* = 0.001). The scores of the positive psychological symptoms were higher than those of the negative psychological symptoms (Table 3).

### 3.3. Comparison of Sleep Quality between Different Genotypes of *CLOCK* (rs1801260)

Given that the PSQI scores presented a skewed distribution, the rank sum test was used to compare sleep quality scores of *CLOCK* (rs1801260) of different genotypes. The results showed differences for the PSQI score (*p* < 0.001), subjective sleep quality (*p* < 0.001), time of falling asleep (*p* < 0.001), sleeping time (*p* < 0.001), sleep efficiency (*p* < 0.001), sleep disorders (*p* = 0.011), and daytime dysfunction (*p* < 0.001), and it can therefore be considered that PSQI scores were different among different genotypes at rs1801260. Using the CC genotype as a reference, post hoc tests were conducted on the scores of the above genotypes showing differences, and the results showed that for the PSQI score, TT was higher than CC (^a^*p* < 0.001); for subjective sleep quality, TT was higher than CC (^b^*p* = 0.05); for time of falling asleep, TT was higher than CC (^c^*p* < 0.001); for sleeping time, TT was higher than CC (^d^*p* = 0.048); for sleep efficiency, TT was higher than CC (^e^*p* < 0.001); and for daytime dysfunction, TT was higher than CC (^f^*p* = 0.05) (Table 4).

### 3.4. Comparison of Sleep Quality among Different Genotypes of *CLOCK* (rs6850524)

Given that the PSQI scores presented a skewed distribution, the rank sum test was used to compare sleep quality scores of *CLOCK* (rs6850524) of different genotypes. The results showed difference for the PSQI score (*p* < 0.001), subjective sleep quality (*p* < 0.001), time of falling asleep (*p* < 0.001), sleeping time (*p* < 0.001), sleep efficiency (*p* < 0.001), sleep disorders (*p* = 0.015), and daytime dysfunction (*p* = 0.001), and it can be considered therefore that PSQI scores were different among different genotypes at rs6850524. Using the CC genotype as a reference, post hoc tests were conducted on the scores of the above genotypes showing differences, and the results showed that for the PSQI score, GG was higher than CC (^a^*p* < 0.001); for subjective sleep quality, GG was higher than CC (^b^*p* = 0.022); for time of falling asleep, GG was higher than CC (^c^*p* < 0.001); for sleeping time, GG was higher than CC (^d^*p* < 0.001); for sleep efficiency, GG was higher than CC (^e^*p* < 0.001); for sleep disorders, GG was higher than CC (^f^*p* < 0.022); and for daytime dysfunction, GG was higher than CC (^g^*p* = 0.004) (Table 5).

### 3.5. Logistic Regression Analysis of Factors Affecting Sleep Disorders

**Figure 1:**
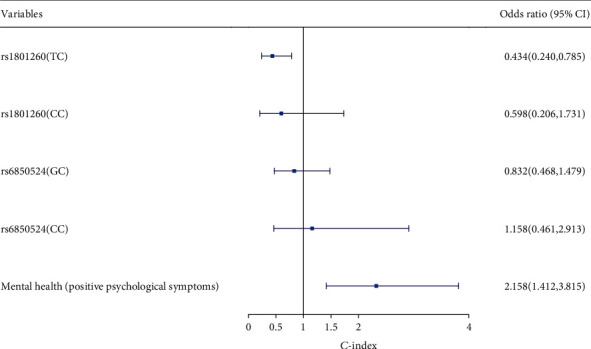
Logistic regression analysis of factors influencing sleep disorders in nurses.

To analyze further the factors affecting sleep quality, the dependent variable was the presence or absence of sleep disorders (0 = negative for sleep disorders and 1 = positive for sleep disorders); *CLOCK* gene polymorphisms (rs1801260, rs6850524) and mental health were taken as independent variables and entered into multiple logistic regression analysis. In the model, rs1801260 (TT) of the *CLOCK* gene was used as the reference group, and rs1801260 (TC) was a protective factor for sleep disorders (OR = 0.434, 95% CI: 0.240–0.785). Positive psychological symptoms are risk factors for sleep disorders (OR = 2.158, 95% CI: 1.412–3.815). It is suggested that the TC genotype at rs1801260 of the *CLOCK* gene and positive psychological symptoms are factors influencing sleep disorder (Figure 1).

### 3.6. Influence of Interaction between Mental Health and *CLOCK* Gene on Sleep Quality

**Figure 2:**
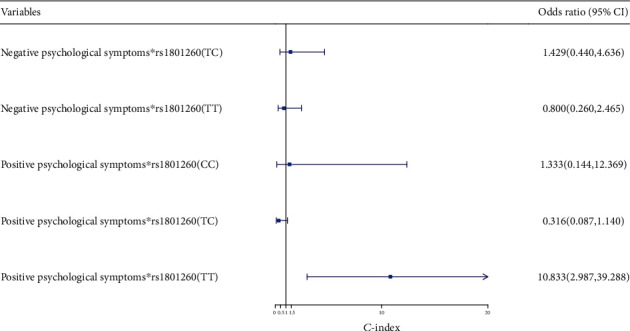
Correlation analysis between mental health∗*CLOCK* gene interaction and sleep disorders. Note: “_∗_” means interaction.

With negative psychological symptoms∗rs1801260 (CC) as the reference, logistic regression analysis was performed on the influence of the interaction between mental health and *CLOCK* gene (rs1801260) on sleep quality. The results showed that the interaction of positive psychological symptoms∗rs1801260 (TT) was a risk factor for sleep disorders (OR = 10.833, 95% CI: 2.987–39.288) (Figure 2).

## 4. Discussion

People spend about one-third of their lives sleeping, as an essential physiological activity to restore the body, consolidate, and integrate the memory. Sleep quality is not only conducive to the recovery of the body to a good state but is also an important prerequisite to ensure the ability to work.

The results of this study found that the incidence of sleep disorders in nurses varied with age, working years, frequency of night shift, and marital status. Nurses aged 26–35 years had the highest incidence of sleep disorders; this may be due to the fact that most nurses aged between 26 and 35 are on the rise in their careers compared with young nurses who have just started work, which leads to greater work pressure. Spending a long time in a state of high pressure at work causes mental tension, which may lead to sleep quality decline and cause sleep problems. On the other hand, with an increase in age, the central nervous system gradually tends to age, and at the same time, the level of hormone secretion will also change significantly, resulting in abnormal metabolic function and even insomnia, anxiety, and other phenomena. A study based on a large sample of people highlighted that, owing to the increase with age of repeated wakefulness in sleep and the significant changes of hormones, the metabolic regulatory function of the human body becomes abnormal, and the sleep quality gradually declines due to the obvious increase of sleep and breathing disorders, insomnia symptoms, depression and anxiety symptoms, and deterioration of general physical condition. Therefore, nurses under the age of 26 have the lowest incidence of sleep disorders, while paradoxically those over the age of 36 may have a relatively low incidence of sleep disorders compared with the 26–35 age group, due to their more stable work and low work pressure. However, the incidence of sleep disorders was higher in nurses with a working age of more than 10 years. Although nurses who have been working for more than 10 years have rich working experience and are skilled, with the passing of their working years, nurses' enthusiasm and freshness for their work gradually decline because of the long-term repetitive work content. This may cause them to have negative emotions, which may affect their sleep. Araghi et al. [42] report that sleep quality has an obvious association with length of service in various occupations and that experienced, skilled workers are more vulnerable to the attention of the leadership and their technical skills lead to higher risk, so that working pressure is greater, which causes insomnia and a decrease in sleep quality.

The incidence of sleep disorder in nurses with shift frequency of >3 times/month was significantly higher than that in nurses with shift frequency < 3 times/month. Shift working has a significant impact on the sleep of workers. Nurses often have to face frequent shift work and have no fixed rest time at night, nor can they ensure adequate sleep. Irregular work and rest time compromise the ability to regulate the body and relieve fatigue. Long and irregular shifts lead to compulsive anxiety, helplessness, imbalance, and other psychological problems among nurses [43, 44]. Zohreh et al. [45] applied the Pittsburgh Sleep Quality Index scale to the study of 160 shift nurses in two hospitals; the results showed that about 50% of shift nurses' sleep disturbance occurred after a shift change. The incidence of sleep disorders among married nurses was also relatively high, which may be due to the heavy workload and long shift hours in the medical industry, resulting in less time for nurses to take care of their family, which may easily lead to conflict between the needs of work and family, resulting in the imbalance between work and life. Those with family responsibilities and economic pressure are prone to anxiety, job burnout, and other adverse psychological states, leading to poor sleep quality [46].

The results of this study found that positive psychological symptoms were a risk factor for sleep disorders, and the PSQI score of the positive psychological symptom group was significantly higher than that of the negative psychological symptom group. Owing to the nature of nursing work, nurses must show patience and care, and frequent shift changes disturb the normal circadian rhythms; living in such a state for a long time may cause female endocrine disorders, anxiety, depression, and other negative emotions, resulting in a decline in the quality of sleep. Logistic regression analysis also suggested that positive psychological symptoms were a risk factor for sleep disorders (OR = 2.158, 95% CI: 1.412-3.815). It has been found that anxiety, depression, and other negative emotions were significantly positively correlated with sleep problems, suggesting that mental health problems seriously affect sleep quality, and the interaction between sleep disorders and low mental health level results in a vicious circle [46]. Therefore, alleviating nurses' negative emotions and improving their mental health state are one of the effective ways to improve nurses' sleep quality [47].

In this study, it was found that the PSQI scores of each genotype and allele at rs1801260 and rs6850524 of the *CLOCK* gene were statistically different. The PSQI scores of the rs1801260 (TT) and rs6850524 (GG) genotypes were higher than those of other genotypes. This suggested that the rs1801260 and rs6850524 loci of *CLOCK* gene might be associated with sleep disorders. Multiple logistic regression analysis showed that the rs1801260 (TC) genotype was a protective factor for sleep disorder when compared with the rs1801260 (TT) genotype (OR = 0.434, 95% CI: 0.240-0.785). The rs1801260 (TT) was a susceptible genotype for sleep disorder. The *CLOCK* gene is first related to the biological clock of eukaryotic gene to be found through research and plays an extremely important role in the regulation and control of organs and tissues restricted by the biological clock, such as the heart, liver, and kidney [48]. The *CLOCK* gene is the most important gene associated with the endogenous molecular circadian clock. It encodes proteins that regulate circadian rhythm. Mutation of the *CLOCK* gene causes changes in the sleep structure and sleep quality. It has been found that the G allele of the *CLOCK* gene rs11932595 was associated with self-reported sleep problems in healthy adults. The polymorphism of the *CLOCK* 3111T/C gene locus was associated with sleep disorders in depressed people: people with TC and CC alleles were more likely to have difficulty falling asleep, early wakening, and poor sleep maintenance [49]. With negative psychological symptoms∗rs1801260 (CC) as the reference, the interaction of positive psychological symptoms∗rs1801260 (TT) was a risk factor for sleep disorders (OR = 10.833, 95% CI: 2.987–39.288). Schuch et al. [50] believe that changes in the *CLOCK* gene coexist with common mental diseases and circadian rhythm disorders. The interaction between *CLOCK* gene rsll932595 and ARNTL gene rsl1824092 was associated with sleep disorders in patients with bipolar disorder [32]. All the above studies have shown that there is a correlation between mental health and the *CLOCK* gene; the interaction between mental health and the *CLOCK* gene can also affect sleep, but the mechanism requires further study.

## 5. Conclusion

The sleep quality of nurses is not only affected by demographic characteristics such as age, working years, frequency of night shift, and marital status but also affected by mental health status and the *CLOCK* gene. The interaction between mental health and *CLOCK* gene polymorphisms was found to be a risk factor for sleep disorders. Therefore, to improve the sleep quality of nurses, it is suggested that hospital managers should appropriately extend the shift cycle, adjust the sleep rhythm of medical staff, relieve work pressure, avoid overwork, and improve negative emotions. The shortcomings and expectations of this study are the following: This study was a cross-sectional study, so causal inference cannot be made. Only 521 surgical nurses were surveyed in this study, so it is difficult to infer the overall situation of nurses owing to the small sample size; at the same time, the sample size for the gene assay was small, only 20% of the nurses with sleep disorder were tested, and the sample size should be increased in the future. At present, the conclusions regarding the relationship between the *CLOCK* gene and mental health are inconsistent. In the future, mental health should be analyzed by various measurement methods, and the relationship between the *CLOCK* gene and mental health should be further discussed. In this study, only the Pittsburgh Sleep Index scale was used to evaluate the sleep of the participants, and the evaluation results were relatively simple. Future studies will use a variety of methods to evaluate sleep quality, such as polysomnography.

## Figures and Tables

**Table 1:** PCR primer sequences.

Primer	Direction	Sequence
*CLOCK* rs1801260	Forward	5′-TCCACGAGTTTCATGAGATCG-3′
	Reverse	5′-GAGGTCATTTCATACGTGACG-3′
*CLOCK* rs6850524	Forward	5′-CCCCAAATACTTGAAGATTA-3′
	Reverse	5′-CTGACACCATCGCTGGTTAA-3′

**Table 2:** Comparison of the incidence of sleep disorders in participants of different demographic characteristics.

Variables		*N*	Sleep disorders *n* (%)	*χ* ^2^	*p*
Age (years)	≤25	267	138 (51.7)	6.971	0.031
	26-35	214	136 (63.6)		
	>35	40	24 (60.0)		
Ethnicity	Han	369	208 (56.4)	0.355	0.551
	Minority	152	90 (59.2)		
Working years	<10 years	312	167 (53.3)	4.284	0.038
	~10 years	209	131 (62.7)		
Educational level	Below bachelor's degree	106	60 (56.6)	0.019	0.890
	Bachelor degree or above	415	238 (57.3)		
Professional titles	Primary	352	192 (54.4)	3.118	0.077
	Intermediate or above	169	106 (62.7)		
Night shift frequency	≤3 times/month	131	39 (29.8)	53.771	<0.001
	>3 times/month	390	259 (66.4)		
Marital status	Single	140	69 (49.3)	4.895	0.027
	Married	381	229 (60.1)		

**Table 3:** Comparison of PSQI scores in participants with different mental health conditions.

Variables	Negative psychological symptoms	Positive psychological symptoms	*Z*	*p*
PSQI score	6.41 ± 3.17	8.47 ± 3.40	-6.651	<0.001
Subjective sleep quality	1.57 ± 0.86	1.94 ± 0.83	-4.943	<0.001
Time of falling asleep	1.22 ± 0.93	1.70 ± 0.95	-5.519	<0.001
Sleeping time	1.39 ± 0.93	1.64 ± 0.97	-3.040	0.002
Sleep efficiency	0.41 ± 0.68	0.67 ± 0.75	-4.435	<0.001
Sleep disorders	0.89 ± 0.63	1.24 ± 0.78	-5.610	<0.001
Hypnotic drugs	0.07 ± 0.27	0.19 ± 0.48	-1.988	0.047
Daytime dysfunction	0.87 ± 0.92	1.14 ± 0.94	-3.520	0.001

**Table 4:** Comparison of sleep quality scores among participants with *CLOCK* gene (rs1801260) (*n* = 120).

	TT	TC	CC	*χ* ^2^	*p*
PSQI score	10.04 ± 3.83^a^	6.61 ± 3.76	6.28 ± 3.41	56.627	<0.001
Subjective sleep quality	1.64 ± 0.92^b^	1.00 ± 0.83	1.17 ± 0.99	30.655	<0.001
Time of falling asleep	2.37 ± 0.74^c^	1.60 ± 0.98	1.39 ± 0.61	55.460	<0.001
Sleeping time	1.82 ± 0.89^d^	1.33 ± 0.99	1.33 ± 0.77	18.632	<0.001
Sleep efficiency	0.94 ± 0.83^e^	0.34 ± 0.76	0.17 ± 0.51	55.881	<0.001
Sleep disorders	1.30 ± 0.69	1.06 ± 0.69	1.00 ± 0.69	9.065	0.011
Hypnotic drugs	0.43 ± 0.81	0.21 ± 0.51	0.17 ± 0.38	5.430	0.066
Daytime dysfunction	1.54 ± 0.91^f^	1.07 ± 0.88	1.06 ± 1.11	19.097	<0.001

Note: the results of post hoc tests, ^a^*p* < 0.001, ^b^*p* = 0.05, ^c^*p* < 0.001, ^d^*p* = 0.048, ^e^*p* < 0.001, and ^f^*p* = 0.05.

**Table 5:** Comparison of sleep quality scores among participants with *CLOCK* gene (rs6850524) (*n* = 120).

	GG	GC	CC	*χ* ^2^	*p*
PSQI score	10.67 ± 3.71^a^	7.14 ± 3.85	6.32 ± 3.02	63.660	<0.001
Subjective sleep quality	1.68 ± 0.90^b^	1.15 ± 0.91	1.21 ± 0.96	22.764	<0.001
Time of falling asleep	2.47 ± 0.72^c^	1.73 ± 0.94	1.61 ± 0.79	53.567	<0.001
Sleeping time	2.07 ± 0.87^d^	1.28 ± 0.91	1.18 ± 0.48	56.549	<0.001
Sleep efficiency	1.17 ± 0.88^e^	0.35 ± 0.61	0.04 ± 0.19	94.696	<0.001
Sleep disorders	1.32 ± 0.67^f^	1.13 ± 0.72	0.96 ± 0.64	8.383	0.015
Hypnotic drugs	0.41 ± 0.80	0.27 ± 0.58	0.36 ± 0.83	1.624	0.444
Daytime dysfunction	1.54 ± 0.91^g^	1.23 ± 0.93	0.96 ± 0.96	13.681	0.001

Note: the result of post hoc tests, ^a^*p* < 0.001, ^b^*p* = 0.022, ^c^*p* < 0.001, ^d^*p* < 0.001, ^e^*p* < 0.001, ^f^*p* = 0.022, and ^g^*p* = 0.004.

## Data Availability

The data used to support the findings of this study are available from the corresponding author upon request.
